# MultitaskProtDB: a database of multitasking proteins

**DOI:** 10.1093/nar/gkt1153

**Published:** 2013-11-16

**Authors:** Sergio Hernández, Gabriela Ferragut, Isaac Amela, JosepAntoni Perez-Pons, Jaume Piñol, Angel Mozo-Villarias, Juan Cedano, Enrique Querol

**Affiliations:** ^1^Departament de Bioquímica i Biologia Molecular, Institut de Biotecnologia i Biomedicina, Universitat Autònoma de Barcelona, Bellaterra, Barcelona 08193, Spain,^2^Laboratorio de Inmunología, Universidad de la República Regional Norte-Salto, Rivera 1350, Salto 50000, Uruguay and ^3^Departament de Medicina Experimental, Institut de Recerca Biomèdica, Universitat de Lleida, Lleida 25198, Spain

## Abstract

We have compiled MultitaskProtDB, available online at http://wallace.uab.es/multitask, to provide a repository where the many multitasking proteins found in the literature can be stored. Multitasking or moonlighting is the capability of some proteins to execute two or more biological functions. Usually, multitasking proteins are experimentally revealed by serendipity. This ability of proteins to perform multitasking functions helps us to understand one of the ways used by cells to perform many complex functions with a limited number of genes. Even so, the study of this phenomenon is complex because, among other things, there is no database of moonlighting proteins. The existence of such a tool facilitates the collection and dissemination of these important data. This work reports the database, MultitaskProtDB, which is designed as a friendly user web page containing >288 multitasking proteins with their NCBI and UniProt accession numbers, canonical and additional biological functions, monomeric/oligomeric states, PDB codes when available and bibliographic references. This database also serves to gain insight into some characteristics of multitasking proteins such as frequencies of the different pairs of functions, phylogenetic conservation and so forth.

## INTRODUCTION

Multitasking or moonlighting refers to those proteins presenting two or more functions performed by a single polypeptide chain. They were initially reported by Wistow and Piatigorsky in the late 1980s when lens crystallins turned out to be the previously known metabolic enzymes (1,2). The term ‘moonlighting’ was coined by Constance Jeffery (3), whereas Joran Piatigorsky proposed ‘gene sharing’ (4). Multitasking proteins present alternative functions that are mostly related to cellular localization, cell type, oligomeric state, concentration of cellular ligands, substrates, cofactors, products or post-translational modifications (3–12). In many cases, a protein uses a combination of these mechanisms to switch between functions. Although some findings suggest involvement of a protein in extra functions, i.e. multitasking proteins can be found in different cellular localizations or in amounts exceeding those required for their canonical function; usually multitasking proteins are experimentally revealed by serendipity. Therefore, any alternative method to identify these proteins would be valuable. In previous works, we have explored the possibility of identifying multitasking proteins using bioinformatics approaches (13) and protein interactomics database information (14). Some authors have suggested that there is a relationship between protein conformational fluctuations and promiscuous functions of proteins, whereas some structurally disordered regions involved in their interaction with different partners are crucial (15,16). Nevertheless, although there are examples of multitasking proteins belonging to the Intrinsically Disordered Protein Class (i.e. p53), in a recent work we found that multitasking proteins are not more prone to belong to the Intrinsically Disordered Proteins (IDP) class than the average (17).

During the development of our previous work aimed at trying to find bioinformatics approaches to predict multitasking proteins, we encountered the difficulty of collecting examples of such proteins because of the lack of a broad database, so the effort to gather the examples was often one of the main challenges. To facilitate the work to researchers interested in the field, we decided to make our set of multitasking proteins freely available as a web database. To our knowledge, a database of multitasking proteins has not yet been compiled. On an extensive data mining, we have found ∼288 proteins elsewhere reported as multitasking proteins, with which we have made a database, named MultitaskProtDB, and designed the corresponding web interface http://wallace.uab.es/multitask/. The database contains information and direct links to all these proteins as well as their accession numbers, species to which they belong, canonical and additional biological functions, PDB codes, if available and the corresponding publications (18–20). Even though the different functions have been called in our database ‘canonical’ and moonlighting*,* this does not involve any biological relevance and merely reflects the historical order of their biological function discovered. The question of which was the first function and which one was lately acquired could be established by evolutionary comparative analysis and our database may help to perform these studies. Probably there are examples of multitasking cases hidden in the literature in which the authors have not recognized this phenomenon or have not bothered to assign their proteins.

## MATERIALS AND METHODS

### Sources of the database

In addition to the examples extracted from the small number of reviews about multitasking proteins (3–12), we have collected >288 multitasking proteins from an inspection of the NCBI PubMed server (19). The literature mining has been performed using the following terms and key words: moonlight proteins; moonlighting proteins; multitask protein; multitasking proteins; moonlight enzymes; moonlighting enzymes; and gene sharing. A number of examples have been found by serendipity from some reviews on protein function, bibliography of sequenced genomes and so forth.

### Design of the database

The database has been created using MySQL. The webserver has been designed with the PHP programming language and assisted by PHPRunner, an application that helps to generate PHP code and to create reports, lists and forms facilitating the development of the important parts of the web. These reports can also be generated using an advanced search engine to allow a more accurate or restricted search. This kind of procedure serves to narrow the search to the subset of proteins to which one really wants to focus the study.

## RESULTS

**Figure 1.**
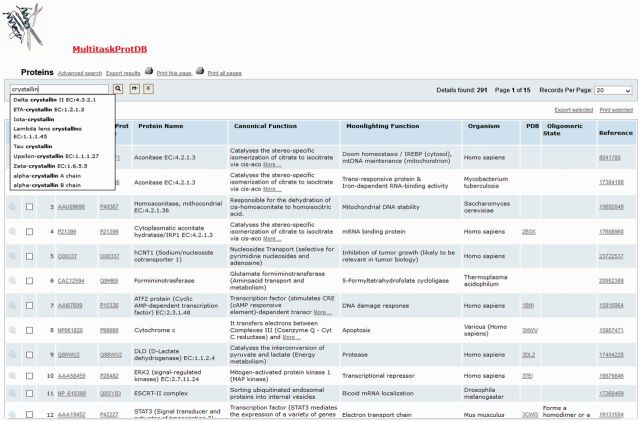
A screenshot of MultitaskProtDB page. Currently, the database contains information ∼288 multitasking proteins that can be easily viewed with the search button and other display facilities. There are several protein characteristics of some multitasking proteins that are not present in the database because no data have been found, especially for PDB structure or oligomeric state. The last column, ‘Reference’, links to the NCBI PubMed article.

On opening the database web page a large table containing 288 entries of multitasking proteins is shown (See Figure 1). It is divided into 15 pages, a maximum of 20 entries for each page, with information on all the collected multitasking proteins. There are 12 columns in the table to characterize each protein. From left to right shows the following: column 1 is a clickable button to see the complete record details. Column 2 allows for entry selection to export and manipulate its contents, if required. Column 3 (ID) indicates the correlative number of the entry in the table. Columns 4 (NCBI Code) and 5 (UniProt Code) show the NCBI and UniProt accession numbers, respectively, which are linked to the corresponding databases information (19,20). Column 6 (Protein Name) displays the protein name and the corresponding Enzyme Commission (EC) number (21). Columns 7 (Canonical Function) and 8 (Moonlighting Function) show the canonical and moonlighting functions, respectively. Column 9 (Organism) indicates the organism in which the moonlighting protein has been identified. Column 10 (PDB) links to the PDB 3D structure of the protein, if available (18). Column 11 (Oligomeric State) indicates the oligomeric depend state of the protein when reported. There are proteins whose multitasking function depends whether they are in mono or oligomeric state. This is the case for one of the major multitasking proteins, Glyceraldehyde 3-phosphate dehydrogenase (GAPDH). Column 12 (Reference) provides a link to the PubMed bibliographic reference (19). Some display, print and search facilities are provided by the web page. Moreover, export of the whole database or the selected entries can be easily done by obtaining a file in different data formats as required by the user for further analysis, such as Excel, Word, Comma Separated Values (CSV) or extensible mark-up language. The database is accessible at http://wallace.uab.es/multitask/.

An overview of the database shows that most disclosed moonlighting proteins present two biological functions. As could be expected, most pairs of functions correspond to different cell compartments when dealing with eukaryotic proteins. When the canonical and the moonlight functions are considered [as broad Gene Ontology descriptors (22), i.e. ‘enzyme and transcription factor; enzyme and cell adhesion’] from the database 30 pairs can be found. The most prevalent pair is ‘enzyme–nucleic acid binding protein’—74 of 288 moonlighting proteins—including in this class transcription factors and nucleic acid binding proteins. Another finding is the lack of integral membrane proteins, which is logical because multitasking proteins usually have each function in different cellular compartments, leading to problems for membrane proteins. Nevertheless, the second prevalent pair-of-functions correspond to an ‘enzyme-adhesion protein’ of pathogen microorganisms (48 of 288 moonlighting proteins). It is a well-known fact that many pathogens use metabolic enzymes that are not integral membrane proteins as adhesion elements to host proteins that require the membrane localization through different mechanisms (23,24). Owing to the high number of cases reported from crystallin proteins the ‘enzyme-structural protein’ pairs are also abundant (30 of 288).

## DISCUSSION

Although several short reviews on moonlighting proteins exist (3–12), they generally only report small number of examples, up to 30–40 at most.

One of the most striking issues of the mammalian (human) genome is the low number of protein-coding genes. To date, the main molecular mechanism used to increase the number of protein isoforms and functions is alternative splicing. However, a less known way to increase the number of protein functions is the existence of multifunctional, multitasking or ‘moonlighting’ proteins. Contrary to splicing, multitasking can be used by microorganisms. For example, a minimal cell like the genera *Mollicutes* or *Mycoplasmas* (which is an experimental objective of the authors too) seems to make extensive use of moonlighting (25,26). We have previously reported that the protein HsdS from *Mycoplasma genitalium*, which was annotated as the DNA binding subunit of the restriction system, is also a cytoskeletal protein (27). As stated by Jeffery (9), current moonlighting proteins ‘appear to be only the tip of the iceberg’.

Predicting multitasking proteins will be useful for researchers when designing a knockout experiment because it could have an off target or side effect with some hidden phenotypic traits. In previous work, we have suggested bioinformatics methods to predict protein multifunctionality (13,14). The MultitaskProtDB database will help researchers to identify protein characteristics and group them to gain insight into protein biological function.

Updates of the database are planned to be done periodically by adding new multitasking proteins as they appear in the literature. These data could help bioinformatics identification of the multitasking proteins and serve as a source of data to create models or validate hypothesis about these proteins. We also wish to ask for the collaboration of those researchers who are involved in these proteins and want to include their published examples. If his/her protein is not listed in the database and they want to include it, please send us an email indicating the specific content they want to appear in each field of the table and the reference.

Another interesting question is the possibility of some multitasking proteins to have more than two different functions and to be hubs in protein–protein interaction networks. A preliminary analysis of a smaller set of multitasking proteins carried out in our laboratory (14) showed that a number of them would correspond to hubs, especially those involved in energy metabolism. In fact, from interactomics it is known than the complexes with more edges (connections) correspond to those of energy metabolism and protein synthesis. However, we have not yet extended the analysis to the present database. In general, moonlighting is also important for the molecular basis of diseases and also for drug discovery because this phenomenon is involved in drug targeting, pharmacodynamics, drug side effects and drug toxicology (28–31).

## FUNDING

Ministerio de Ciencia y Tecnología de Espanya [BIO2007-67904-C02-01, BFU2010-22209-C02-01]; Centre de Referència de R+D de Biotecnologia de la Generalitat de Catalunya; La Marató de TV3 [101930/31/32/33]; Comisión Coordinadora del Interior de Uruguay. The English of this manuscript has been corrected by Ms Lynn Strother. Funding for open access charge: [BIO2007-67904-C02-01 and BFU2010-22209-C02-01].

*Conflict of interest statement*. None declared.
